# Potential molecular mechanisms underlying muscle fatigue mediated by reactive oxygen and nitrogen species

**DOI:** 10.3389/fphys.2015.00239

**Published:** 2015-09-01

**Authors:** Edward P. Debold

**Affiliations:** Department of Kinesiology, University of MassachusettsAmherst, MA, USA

**Keywords:** muscle, fatigue, reactive oxygen species, myosin, troponin, tropomyosin

## Abstract

Intense contractile activity causes a dramatic decline in the force and velocity generating capacity of skeletal muscle within a few minutes, a phenomenon that characterizes fatigue. Much of the research effort has focused on how elevated levels of the metabolites of ATP hydrolysis might inhibit the function of the contractile proteins. However, there is now growing evidence that elevated levels of reactive oxygen and nitrogen species (ROS/RNS), which also accumulate in the myoplasm during fatigue, also play a causative role in this type of fatigue. The most compelling evidence comes from observations demonstrating that pre-treatment of intact muscle with a ROS scavenger can significantly attenuate the development of fatigue. A clear advantage of this line of inquiry is that the molecular targets and protein modifications of some of the ROS scavengers are well-characterized enabling researchers to begin to identify potential regions and even specific amino acid residues modified during fatigue. Combining this knowledge with assessments of contractile properties from the whole muscle level down to the dynamic motions within specific contractile proteins enable the linking of the structural modifications to the functional impacts, using advanced chemical and biophysical techniques. Based on this approach at least two areas are beginning emerge as potentially important sites, the regulatory protein troponin and the actin binding region of myosin. This review highlights some of these recent efforts which have the potential to offer uniquely precise information on the underlying molecular basis of fatigue. This work may also have implications beyond muscle fatigue as ROS/RNS mediated protein modifications are also thought to play a role in the loss of muscle function with aging and in some acute pathologies like cardiac arrest and ischemia.

## Introduction

Intense exercise can be sustained for only a few minutes before the force and motion generating capacity of skeletal muscle is severely compromised; a phenomenon which defines fatigue (Westerblad et al., 1991; Fitts, 1994; Allen, 2009). Understanding the mechanisms through which this occurs is important to our basic understanding of muscle but also for the development of therapies to improve the physical function of the elderly and chronically ill, so debilitated by fatigue. The extent and rate of fatigue from intense exercise is dependent on numerous factors including the intensity of contraction as well as the rate and duration of stimulation (Fitts, 1994). In humans there are many potential sites of failure and many transient intracellular biochemical changes, including the accumulation of metabolites of ATP hydrolysis (Fitts, 1994). The role of accumulating metabolites has been particularly well-studied, detailed in other reviews (Westerblad et al., 1991; Fitts, 1994, 1996; Cooke, 2007; Enoka and Duchateau, 2007; Allen et al., 2008; Allen, 2009; Debold, 2012). Although less well-studied, there is growing evidence that elevated levels of reactive oxygen and nitrogen species (ROS/RNS) accumulate during intense contractile activity and play a causative role in the fatigue process (Westerblad and Allen, 2011).

**Figure 1:**
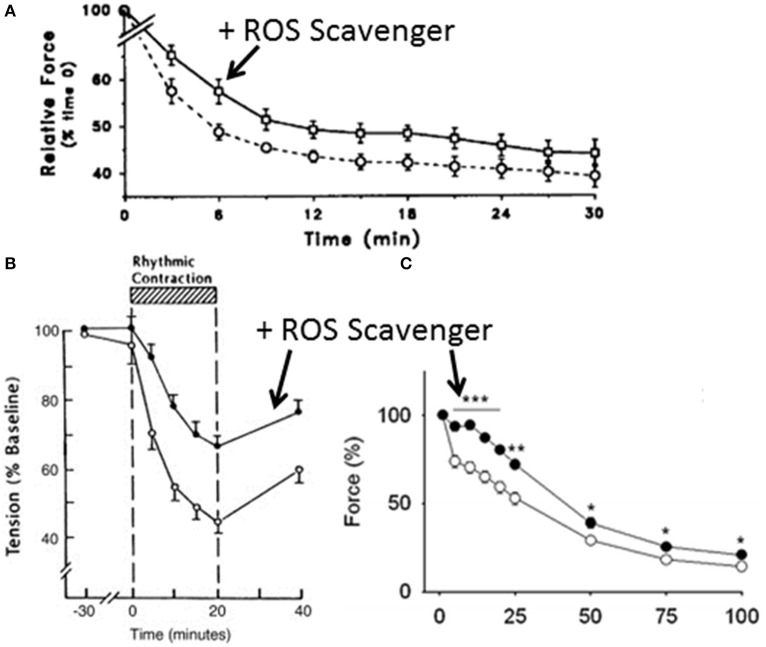
**Effect of reactive oxygen species scavengers on fatigue**. **(A)** Effect of pretreatment with ROS scavenger, *N*-acetylcysteine (circles) vs. control (squares) on the fatigue profile of the tibialis anterior muscle in humans stimulated at 10 Hz with a surface electrode. The drop in force was significant under both conditions but the force was greater with *N*-acetylcysteine treatment. Reprinted from Reid et al. (1994) with rights and permission from American Society for Clinical Investigation. **(B)** Effect of the *N*-acetylcysteine on the development of diaphragm fatigue in an anesthetized intact rabbit preparation. Reprinted from Shindoh et al. (1990) with permission. **(C)** Effect of *N*-acetylcysteine on the development of fatigue in isolated mouse extensor digitorum longus muscle. Reprinted from Katz et al. (2014) with permission. ^*^, ^**^ and ^***^ indicate significantly different from corresponding control value (open circles) at *p* < 0.05, *p* < 0.01, and *p* < 0.001 respectively.

Some of the most compelling evidence comes from the observation that pre-treatment with exogenous ROS scavengers attenuates the rate and extent of fatigue in intact muscle (see Figure 1). While this is most pronounced in isolated muscle preparations (Shindoh et al., 1990; Moopanar and Allen, 2005, 2006) where the *ex vivo* nature of the preparations might augment the level of ROS (Halliwell, 2014), it has also been observed in exercising humans (Reid et al., 1994; Medved et al., 2004; Ferreira et al., 2011; Slattery et al., 2014). The preventative effect of ROS scavengers is most obvious in humans when fatigue is induced with low stimulation frequencies (Reid et al., 1994), but it is also dependent on the variant of ROS scavenger (Hernández et al., 2012). Thus, there is evidence that ROS/RNS scavengers can delay fatigue and that the accumulation of ROS is linked to the onset and extent of fatigue, which provide compelling support for a causative role. The molecular mechanisms underlying these effects remain unclear but in the last 5–10 years several research groups have made significant advances that have identified specific contractile proteins modified by ROS/RNS and their impact on molecular function (Callahan et al., 2001; Moopanar and Allen, 2005, 2006; Prochniewicz et al., 2008; Dutka et al., 2011; Klein et al., 2011; Mollica et al., 2012; Moen et al., 2014b; Cheng et al., 2015). This review focuses on these recent efforts that highlight the potential molecular mechanism of ROS/RNS mediated fatigue.

## What are ROS/RNS and how do they affect muscle function?

ROS/RNS are chemically reactive molecules making them capable of transiently or permanently damaging cell membranes and proteins (Moen et al., 2014b). The most important species of ROS/RNS for muscle contraction appear to be hydrogen peroxide (H_2_O_2_), superoxide (${\text{O}}_{2}^{-.}$) as well as nitric oxide which is a precursor to the RNS peroxynitrite (ONOO^−^), and involved in the S-Nitrosylation of proteins (Reid, 2001a). ROS were originally thought to be primarily generated in the mitochondria as a by-product of oxidative phosphorylation, however more recent findings suggest that the amount generated in the mitochondria is likely quite low, with an upper estimate of ~0.15% (St-Pierre et al., 2002). Instead newer measurements suggest that the primary source of ROS in the cytosol is generated by membrane bound NADPH oxidases (Sakellariou et al., 2011, 2013). For a detailed description of ROS/RNS generating reactions the reader is directed to several excellent reviews (Reid, 2001b; Allen et al., 2008; Powers et al., 2011). Regardless of the source, free radicals are continually produced in muscle cells. At rest, low levels are generated and thought to play an important role in maintaining normal muscle function (Reid, 2001b). In fact, when levels of ROS are low, as might occur at rest or even in the early stages of fatigue, they appear to enhance not inhibit force production (Reid, 2001b) and may even help to delay the onset of fatigue (Mollica et al., 2012). However, as contraction intensifies and/or becomes prolonged, ROS/RNS become elevated and are thought to inhibit proper function of muscle proteins (Reid, 2001a). Consistent with this idea, at higher concentrations, certain ROS inhibit contractile function in isolated muscle fibers (Andrade et al., 1998). When excessively elevated, the reactive nature of ROS/RNS mean that they can induce structural modifications to contractile proteins at cysteine and methionine residues, including for example the formation of disulfide bridges between neighboring cysteine residues either within or between proteins. These modifications may be benign or could depress the function of key contractile proteins. Therefore, goal of much of the recent research in this area has focused on linking loss of contractile protein function to a specific structural alteration; recent advances in biochemical and biophysical techniques are making this a tractable problem.

Skeletal muscle fatigue from intense contractile activity could result from the failure anywhere along the muscle activation pathway, from the firing of neurons in the motor cortex to the generation of force and motion by an actomyosin cross-bridge. A wealth of evidence suggests that this type of fatigue is primarily due to a failure that is distal to the central nervous system (Merton, 1954; Cady et al., 1989; Westerblad et al., 1991). Thus, it is thought that the mechanisms that underlie the role for ROS/RNS in fatigue are also mediated distal to the neuromuscular junction (Westerblad and Allen, 2011). Based on the idea that fatigue originates peripherally there are four key sites at which ROS/RNS mediated fatigue could potentially develop (Westerblad and Allen, 2011): (1) compromised ability to depolarize the sarcolemma; (2) reduction in the amount of Ca^++^ released from the sarcoplasmic reticulum (SR); (3) a decrease in the Ca^++^-sensitivity of the thin filament; and (4) a direct effect on myosin's ability to bind to and translocate an actin filament.

## Do ROS contribute to fatigue by affecting membrane excitability and/or Ca^++^ release from the SR?

A decrease in membrane excitability is one potential mechanism, and based on observations in intact fibers this is believed to occur during fatigue (Juel, 1988), with the effect most pronounced deep within the t-tubules (Balog et al., 1994). Presumably this results from the inability to restore the Na^+^/K^+^ balance with repeated contractions (Juel, 1988) due to ROS modifications of the Na^+^/K^+^ pump acting to inhibit its function (McKenna et al., 2006). Interestingly, pretreatment of humans with *N*-acetylcysteine (NAC) (a thiol containing compound (Ferreira and Reid, 2008) that spurs the resynthesize glutathione, a major endogenous antioxidant) increased time to fatigue at 92% of VO_2_ peak by ~25% (McKenna et al., 2006). Subsequent biochemical analysis of muscle biopsies revealed that NAC attenuated the decline Na^+^/K^+^ pump activity, suggesting that this type of fatigue may involve ROS/RNS modification of proteins responsible for maintaining membrane excitability.

However, a distinctly different conclusion was reached by authors using intact fibers that were exposed to elevated levels of exogenous H_2_O_2_. These findings consistently show that elevated H_2_O_2_ decreases isometric force but not the intracellular [Ca^++^] (Andrade et al., 1998, 2001), suggesting that the signal to the SR to release Ca^++^ and thus membrane excitability is not compromised by ROS in these preparations. Part of the discrepancy in the findings may be due to different stimulation rates employed since the experiments on isolated preparations typically show little or no effect on intracellular Ca^++^-levels with fatigue typically stimulate the muscle at 40 Hz or greater, a rate at which ROS scavengers have little effect on fatigue (Ferreira and Reid, 2008). Another issue may be the timing of the exposure to the oxidants as it has been shown that different regions of the contractile proteins are accessible based on the level of Ca^++^-activation (Gross and Lehman, 2013). Or the discrepancy may be related to the use of exogenous H_2_O_2_, which has been hypothesized to exert different effects than those generated endogenously (Forman, 2007).

If membrane excitability were compromised by fatigue it should translate into a weaker signal activating the voltage sensitive receptors of the t-tubules (DHPR) and in turn less release of Ca^++^ by the ryanodine receptors (RyR), causing less activation of the thin filament and thus less cross-bridge formation and force generation (Balog and Fitts, 1996). Support for this notion comes from observations in mechanically skinned fibers in which fatigue, accelerated by higher temperatures, was mitigated by the superoxide scavenger Tiron (van der Poel and Stephenson, 2007). Consistent with this observation studies utilizing vesicles with RYR channels have demonstrated that this channel can be oxidized and that this can affect its ability to release Ca^++^ by increasing their sensitivity to Ca^++^-induced-Ca^++^-release (CICR) (Marengo et al., 1998). Likewise, exposure of skinned muscle fibers to H_2_O_2_ can also increase CICR. Furthermore, NO has been shown to exert regulatory effects on the RYR channels in skeletal muscle (Sun et al., 2001, 2003). Despite these modifications, exposure of isolated intact muscle preparations to exogenous ROS have generally demonstrated insignificant effects on Ca^++^ released from the SR in response to an action potential (Lamb and Posterino, 2003; Posterino et al., 2003), even at higher temperatures(Place et al., 2009). This suggests that Ca^++^ release is not susceptible to ROS when activated via the nerve whereas it is when stimulated artificially in skinned fibers. Further support for this notion comes from observations showing that the ROS scavenger, Tiron, significantly attenuates the development of fatigue in intact fibers without altering the intracellular [Ca^++^] (Moopanar and Allen, 2005). Therefore, despite the findings in skinned muscle fibers most current evidence in intact preparations suggests that ROS/RNS mediated fatigue is not due to a loss of membrane excitability or an inability to release Ca^++^ but rather to an effect downstream of Ca^++^ release (Westerblad and Allen, 2011).

## Does ROS contribute to fatigue by decreasing myofilament Ca^++^-sensitivity?

During normal muscle activation Ca^++^ release from the SR is followed by a series of complex molecular events that ultimately lead to tropomyosin moving over the surface of actin to reveal the myosin binding sites (Gordon et al., 2000). During fatigue disruption of any one of these steps would disrupt thin filament activation leading a decrease in Ca^++^-sensitivity. These steps include the binding of Ca^++^ to the calcium binding subunit of troponin (TnC), which triggers the c-terminal domain of the inhibitory subunit of Tn (TnI) to retract from its binding site on actin (Takeda et al., 2003), that allows tropomyosin (Tm) to move further into the groove in actin making the myosin binding sites more accessible (Gordon et al., 2000). In the most widely accepted model of thin filament activation Tm is believed be in a dynamic equilibrium among three positions on actin (Galinska-Rakoczy et al., 2008) with the filament only fully activated when a myosin molecule is strongly bound to actin (McKillop and Geeves, 1993). This is required because the strong-binding of myosin moves Tm a further distance away from the myosin binding sites allowing for neighboring myosins to strongly bind to the filament (Galinska-Rakoczy et al., 2008). Therefore, ROS/RNS could elicit fatigue by either altering Tn's ability to bind Ca^++^ and communicate this signal to its other subunits and Tm, and/or by disrupting myosin's ability to strongly bind to actin. This effect may be particularly relevant because in the latter stages of fatigue, through mechanisms independent of ROS, Ca^++^ release from the SR is compromised (Lee et al., 1991) resulting in a lower intracellular level of Ca^++^ with any given level of stimulation.

Some of the earliest efforts, in chemically skinned cardiac muscle fibers, found that exposure to elevated levels of superoxide had a large effect on maximal force but did not reduce Ca^++^-sensitivity (MacFarlane and Miller, 1992). Similarly, exposure of rabbit diaphragm fibers to superoxide reduced maximal force but did not change the concentration of Ca^++^ required to reach half maximal force (pCa_50_) (Darnley et al., 2001). Skinned single skeletal muscle fibers to respond similarly to elevated ROS, seeing a decrease in maximal isometric force with no change in Ca^++^-sensitivity (van der Poel and Stephenson, 2002). Although this study was focused on determining the effects of elevated temperatures on contractile function, the findings are relevant to the role of ROS in muscle fatigue because they indicated that the depression in function at higher temperatures was due to elevated levels of superoxide. Consistent with this idea, the depression in maximal isometric force could be reversed by the disulphide reducing agent dithiotheritol (DTT) (van der Poel and Stephenson, 2002), which is similar to what is seen with fatigue (Moopanar and Allen, 2005). Collectively these findings suggest that ROS have a pronounced, depressive effect on maximal isometric force and little impact on Ca^++^-sensitivity. Thus, these findings point toward ROS potentially having a direct effect on actomyosin and not the regulatory proteins troponin and tropomyosin.

**Figure 2:**
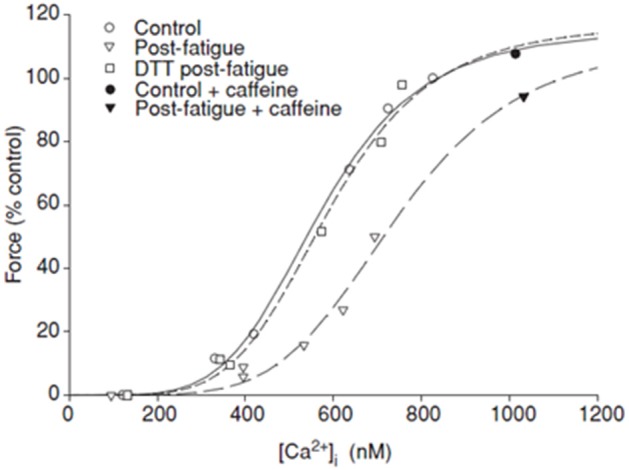
**ROS mediated fatigue and Ca^++^-sensitivity**. Force-calcium relation plotted during fatigue of intact mouse flexor brevis muscle fibers stimulated at 100 Hz, at 37°C. Force expressed relative to rested value under control conditions. Fatigue under control conditions produced a strong rightward shift in the relation and depressed maximal force. Maximal force but not Ca^++^-sensitivity was restored with caffeine. Treatment with dithiothreitol (DTT) restored Ca^++^-sensitivity. Reprinted from Moopanar and Allen (2006) with permission from John Wiley and Sons publishing company.

However, these findings stand in stark contrast to the results from intact muscle preparations which have consistently observed that ROS decreases Ca^++^-sensitivity while exerting minimal effects on maximal isometric force (Andrade et al., 1998, 2001; Moopanar and Allen, 2005, 2006). For example, elevated levels of H_2_O_2_ produced a pronounced rightward shift in the force-calcium relation in intact muscle fibers without affecting the intracellular Ca^++^ concentration (Figure 2), suggesting that the H_2_O_2_ decreased the Ca^++^-sensitivity of the myofilaments (Andrade et al., 1998). This work was confirmed and extended in a more recent where intact muscle was fatigued in the presence of DTT (Moopanar and Allen, 2006). In this study living mouse fast muscle fibers were stimulated to fatigue while simultaneously measuring isometric force and intracellular levels of Ca^++^, enabling the ability to directly determine the force-calcium relationship in an intact fiber during fatigue. Consistent with prior experiments in intact muscle, Ca^++^ release was compromised by fatigue but this was not reversed by DTT, indicating no role for ROS. Ca^++^-sensitivity however was depressed and was fully reversed by DTT. While subsequent findings suggested that iron leeching off the stimulating electrode in these experiments likely drove the ROS concentration higher than originally reported (Reardon and Allen, 2009a,b) it remains clear that elevated levels of ROS mediated the depressive effects on force. Therefore, these findings suggest that elevated levels of ROS decrease the Ca^++^-sensitivity of the myofilaments and that this may be the primary mechanism through which ROS induces fatigue in intact muscle.

It is not clear why the results from skinned muscle fibers differ so strongly from those using intact muscle fibers. While it can be argued that intact fibers, which can be exposed to a fatiguing protocol, represent the more physiologically relevant findings, a careful delineation of the differences between these findings may provide key insights into the mechanisms underlying ROS induced muscle fatigue. Along this line of thought, a recent report focused on prolonged low-frequency force depression observed that both the SR Ca^++^-release and myofilament sensitivity can be altered by fatiguing stimulation in intact fibers (Cheng et al., 2015). To explain these findings they suggested a model where the mechanism of ROS mediated fatigue is dependent on the stimulation protocol, with one type of protocol superoxide is generated in the mitochondria and owing to its proximity and possible physical connections to the SR (Boncompagni et al., 2009) directly affects the DHP and RyR channels, and as a result reduces Ca^++^ release, but has no effect on Ca^++^-sensitivity. In contrast, other types of fatigue protocols, and exposures to exogenous ROS/RNS, result in a more broad cytosolic elevation of ROS (e.g., exogenous H_2_O_2_ exposure) that modifies the thick and thin filaments of the sarcomere, leading to a decrease in Ca^++^-sensitivity.

Since DTT targets disulphide linkages the findings of Moopanar and Allen (2006) suggest that these types of modifications are driving the ROS mediated fatigue, but it is unclear which of the contractile proteins involved determining the Ca^++^-sensitivity are modified by the ROS. However, related work in diaphragm muscle fatigued under hypoxic conditions may offer insight (de Paula Brotto et al., 2001). In response to fatiguing stimulation there was evidence of TnI and TnC degradation, both of which are integral to proper thin filament activation. The loss in force during fatigue was attributed to the cleavage of these subunits of Tn which the authors speculated resulted from ROS modification that targeted them for proteolysis (de Paula Brotto et al., 2001). The authors also speculated that although hypoxia might generate a greater and more diverse pool of ROS that the same ROS might also appear during physiological fatigue because muscle is thought to become hypoxic to some extent during fatigue even under normoxic conditions (Babcock et al., 1995).

Alternatively fatigue may not lead to cleavage of TnI but rather might induce post-translational modifications of Tn/Tm. In support of this notion a recent study focused on identifying the mechanisms of the increased Ca^++^-sensitivity following a treatment that oxidizes cysteine residues (disulphide 2,2′-dithiodipyridine or DTDP) found that the effects could be attributed to the addition of a glutathione to a specific cysteine residue (Cys133) in TnI (Mollica et al., 2012). This residue is in a crucial mobile region of TnI that switches between being bound to actin (low Ca^++^) and bound to a hydrophobic patch on the N-lobe of TnC (High Ca^++^) (Takeda et al., 2003). Therefore, this modification likely would lead to this region more readily associating with TnC and therefore could explain the increased Ca^++^-sensitivity under the conditions tested. This effect appears to increase with exercise and thus the authors suggested it may help delay the onset of, rather than contribute to, fatigue. Thus, this effect is the opposite of the depressive effects described above but highlights that the regulatory proteins can be post-translationally modified in response to exercise. It would be interesting to determine if the modification is lost as fatigue becomes more severe or if other alterations that lead to depressed Ca^++^-sensitivity appear in the same crucial region of TnI.

Yet another possible explanation for these observations would be the oxidation-induced dissociation of TnC from the troponin complex and thus the thin filament. This notion is supported by evidence showing that oxidation of the Cys 98 residue of the D/E helix of TnC reduces its affinity for the thin filament and the effect is readily reversed by exposure to DTT (Pinto et al., 2011). It will be informative to understand the mechanisms underlying this event in more detail and how it might impact fatigue in future work.

Overall these data demonstrate strong evidence that the ROS/RNS mediated fatigue could involve alterations in the structure and function of the muscle regulatory proteins that is consistent with the observed decrease in Ca^++^ sensitivity.

## Is myosin's function affected by ROS/RNS during fatigue?

The skinned fibers studies demonstrating that elevated levels of ROS/RNS decrease maximal isometric force but not Ca^++^sensitivity suggest that under these conditions ROS/RNS may act by modifying myosin and/or actin. Such targets and modifications have yet to be thoroughly investigated but some recent work focused on understanding the broad effects of oxidative stress on muscle have highlighted that myosin may have redox sensitive residues in the actomyosin interface (Prochniewicz et al., 2008; Klein et al., 2011; Moen et al., 2014a,b) that affect its function. Consistent with others, this group has shown that non-physiological concentrations of H_2_O_2_ (5–50 mM) can reduce the maximal isometric force, maximal ATPase rate and Ca^++^ -sensitivity of chemically skinned skeletal muscle fibers (Prochniewicz et al., 2008). Electron paramagnetic resonance labeling experiments revealed that the treatment increases the fraction of myosin heads in the strongly bound state in relaxed conditions (i.e., low Ca^++^). With more heads in a strongly bound state at rest, less heads will be able to go through the weak-to-strong force generating transition upon activation, and thus this may explain the decrease in maximal isometric force in the muscle fibers in response to H_2_O_2_. Further, analysis using mass spectrometry showed the addition of multiple oxygen atoms on both myosin's heavy and light chains, which occurred exclusively at methionine residues. This provides a link between the loss of force generation with a modification to a specific residue on myosin. In a follow up study, using myosin expressed in *Dictyostelium Discoideum* (*Dicty*), the authors demonstrated that most of H_2_O_2_'s effects on myosin function can be attributed to oxidation at a single methionine residue (M394) in the actin-binding interface (Klein et al., 2011). This hypothesis is further supported by more recent work demonstrating that the depressive effects of oxidation at this site can be completely reversed by exposure to methionine sulfoxide reductase targeting the M394 site (Moen et al., 2014a). Interestingly in human skeletal muscle myosin this residue is a Cys not a Met, but the effects of oxidation at this residue when it is changed to a Cys in the *Dicty* construct are nearly identical to those when M394 is oxidized (Moen et al., 2014a). Thus, this is an interesting line of experiments and highlights that myosin can be modified by ROS species which are elevated during fatigue, and that the functional differences are attributable to the modification at a single residue in the actomyosin interface. It will be important to determine if the modification also happens in human skeletal muscle during fatigue and to demonstrate that it contributes to the depressive effects seen during fatigue. This would be particularly exciting because presumably it could be readily reversed.

### Conflict of interest statement

The author declares that the research was conducted in the absence of any commercial or financial relationships that could be construed as a potential conflict of interest.
